# Mining and Analysis of Salt Tolerance Genes in Maize at the Seedling Stage

**DOI:** 10.3390/cimb48040423

**Published:** 2026-04-20

**Authors:** Zhenping Ren, Zelong Zhuang, Jianwen Bian, Wanling Ta, Xiaojia Hao, Lei Zhang, Yunling Peng

**Affiliations:** 1 College of Agronomy, Gansu Agricultural University, Lanzhou 730070, China; renzp1003@163.com (Z.R.); zhuangzl3314@163.com (Z.Z.); bjwen1018@163.com (J.B.); kellytwl@163.com (W.T.); haoxj772@163.com (X.H.); 18919106150@163.com (L.Z.); 2 State Key Laboratory of Aridland Crop Science, Gansu Agricultural University, Lanzhou 730070, China; 3 Seed Industry Research Institute of Gansu Provincial Universities, Lanzhou 730070, China

**Keywords:** maize, salt stress, BSA-seq, transcriptome, ion equilibrium, reactive oxygen species

## Abstract

Salt stress represents a significant abiotic stress factor that adversely affects plant growth and development. It directly inhibits both vegetative and reproductive growth, resulting in substantial reductions in crop yield and quality. Consequently, the identification of salt tolerance genes and the elucidation of their underlying molecular mechanisms are crucial for improving crop salt tolerance and ensuring agricultural productivity. To investigate the molecular basis underlying differential salt tolerance between Zheng58 and PH4CV, we employed pooled sequencing (BSA-seq) using extreme phenotypic individuals from their F_2_ population and conducted a comparative transcriptome analysis at the seedling stage of the two genotypes. Phenotypic, physiological, biochemical, and ion content analyses revealed that Zheng58 exhibited significantly superior performance compared to PH4CV under salt stress conditions. BSA-seq analysis identified six genomic regions associated with salt tolerance, encompassing a total of 391 genes. Functional annotation enabled the screening of 151 candidate genes potentially involved in salt stress responses. Transcriptome profiling indicated that differentially expressed genes were significantly enriched in biological processes, particularly plant hormone signal transduction and MAPK signaling pathways. Integrating BSA-seq and transcriptome data, key candidate gene *ZmACC2* (*Zm00001eb419400*) was identified as potentially involved in the regulation of salt tolerance in maize. This gene may modulate Na^+^/K^+^/Ca^2+^ homeostasis and reactive oxygen species metabolism through defense responses mediated by ethylene (ETH) and hydrogen peroxide, as well as through ion homeostasis regulatory pathways. This study provides valuable candidate genes and a theoretical foundation for further dissection of the molecular mechanisms governing salt tolerance in maize.

## 1. Introduction

Maize (*Zea mays* L.) is one of the world’s three major cereal crops, providing sustenance for nearly 6 billion people. The stability of its production is of critical importance for ensuring global food supply and meeting growing demand [1]. However, under the pressures of global climate change and water scarcity, extensive maize-producing regions are increasingly threatened by soil salinization [2]. This phenomenon can be categorized into three major types based on the dominant salt composition: chloride-type (mainly NaCl), sulfate-type (mainly Na_2_SO_4_ and MgSO_4_), and carbonate-type (mainly Na_2_CO_3_ and NaHCO_3_) [3,4]. Chloride-type salinity is the most common and widely distributed, especially in coastal regions, arid inland basins, and irrigated farmlands suffering from secondary salinization [5,6]. Sulfate-type salinity often occurs in continental saline soils under moderate leaching conditions, while carbonate-type salinity is typically associated with alkaline soils in semiarid regions [7,8]. Considering that chloride-type salinity dominates the majority of salt-affected agricultural lands worldwide and imposes strong osmotic and specific ionic stresses on plants, NaCl was selected as the stress agent in this study to represent the most environmentally relevant scenario [9]. As a salt-sensitive species, maize experiences significant inhibition of growth and development under high salinity conditions, often leading to substantial yield losses. Without the implementation of effective strategies to address this challenge, agricultural sustainability and global food security may be severely compromised. Research indicates that plants exposed to high-salt environments experience dual stresses—osmotic and ionic—and concurrently generate reactive oxygen species (ROS) [10]. Maize varieties exhibit differential salt tolerance: salt-tolerant genotypes maintain elevated antioxidant enzyme activity, effectively scavenging ROS and restricting Na^+^ uptake and translocation to aerial tissues, thereby mitigating salt damage [11]. Conversely, salt-sensitive genotypes display compromised antioxidant capacity, leading to excessive ROS accumulation, which disrupts physiological functions and impairs primary and secondary metabolism. This ultimately results in severe growth inhibition and substantial reductions in agricultural productivity [12]. Therefore, understanding the physiological and biochemical mechanisms underlying maize adaptation to salt stress, as well as associated changes in ion homeostasis, is essential for the development of salt-tolerant maize varieties [13].

In recent years, significant advances have been achieved in identifying salt tolerance genes and elucidating the mechanisms conferring salt stress tolerance in crops through integrated approaches combining transcriptomics and genetic mapping [14,15,16,17,18,19,20]. Xu et al. demonstrated that under 300 mmol/L NaCl treatment, a salt-tolerant variety, Baiyan2, exhibited significant upregulation of genes involved in energy-consuming and biosynthetic pathways—such as glycolysis, starch, and glucose metabolism—whereas these genes were markedly downregulated in the salt-sensitive variety Baiyan5. Furthermore, Baiyan2 maintained ion homeostasis by modulating the expression of Na^+^/K^+^ transporter genes, resulting in higher K^+^ content and lower Na^+^ accumulation [21]. Ding et al. compared the salt-tolerant genotype SHEN5003 with the sensitive genotype YZ26 and observed that under salt stress, SHEN5003 displayed superior biomass, SPAD values, and K^+^/Na^+^ ratios. Transcriptomic analysis revealed that its enhanced tolerance was associated with the differential regulation of genes involved in plasma membrane homeostasis, including H-ATPase, HKT1, and HAKs, as well as ROS scavenging genes such as CAT2. Additionally, *ZmSWEET1b* was found to exert a positive regulatory role in salt-tolerant materials [22]. Wang et al. reported that under saline conditions, the salt-tolerant variety L87 exhibited significantly higher activities of superoxide dismutase (SOD), peroxidase (POD), and catalase (CAT) compared to the sensitive variety L29, accompanied by reduced ROS accumulation. Specific expression profiling identified 1856 upregulated genes in L87, including key components of ABA signaling such as SnRK2, along with multiple members of the SOD, POD, and CAT gene families, collectively contributing to enhanced stress resilience [23]. In the realm of genetic mapping, Gao et al. employed QTL-seq and BSA-seq to identify 11 candidate salt-tolerance genes within a 5.24 Mb region on chromosome 4 in a sea-rice SR86 hybrid population [24]. Zhu et al. applied BSA-seq in an F_2_ extreme population and identified 106 candidate regions and 77 putative genes, ultimately pinpointing two critical salt-tolerance genes, *Zm00001d037181* and *Zm00001d053925*, in the highly tolerant AS5 lineage [25]. Collectively, these studies have uncovered the molecular and physiological bases of salt tolerance from multiple perspectives—including biochemical responses, transcriptional regulation, and genetic architecture—providing valuable genetic resources and a robust theoretical foundation for the breeding of salt-tolerant crop varieties [26,27].

This study utilized the salt-tolerant variety Zheng58 and the salt-sensitive variety PH4CV, previously identified by the research group [28], as experimental materials to systematically investigate the molecular mechanisms underlying their differential salt tolerance. BSA-seq was performed on two phenotypic extreme pools derived from an F_2_ population to identify candidate genomic regions, followed by functional annotation of genes within these intervals. Subsequently, transcriptome sequencing was conducted to identify differentially expressed genes (DEGs) in response to salt stress, with functional characterization through Gene Ontology (GO) enrichment and Kyoto Encyclopedia of Genes and Genomes (KEGG) pathway analyses. Integrating the BSA-seq and transcriptomic data, 32 candidate genes strongly associated with salt tolerance were screened out. The expression profiles of these candidate genes were further validated using quantitative real-time PCR (RT-qPCR), and the key candidate gene *ZmACC2* (1-aminocyclopropane-1-carboxylate synthase) closely related to maize salt tolerance was ultimately determined. This gene helps to deepen the understanding of the physiological and molecular mechanisms of maize salt tolerance, enriches the available genetic resources, and has potential application value in breeding programs aimed at improving maize salt tolerance.

## 2. Materials and Methods

### 2.1. Plant Material and Growth Conditions

The salt-tolerant variety Zheng58 and the salt-sensitive variety PH4CV were crossed to generate the F_1_ generation, which was subsequently self-pollinated to produce the F_2_ population, thereby completing the construction of the genetic mapping population. At the same time, there were salt-tolerant variety Qi319, salt-sensitive variety N192, as well as varieties Mo17 and B73. Both varieties and the F_2_ population were provided by the Maize Research Group, College of Agronomy, Gansu Agricultural University, and served as experimental materials for the salt tolerance study.

In this study, salt stress was simulated under controlled conditions using sodium chloride (NaCl) treatment to mimic saline environments [29]. The experiment included two treatment conditions—distilled water (control) and 180 mmol/L NaCl—applied to both genotypes, resulting in four treatment groups: Zheng58 + distilled water (ZCK), Zheng58 + 180 mmol/L NaCl (ZN), PH4CV + distilled water (PCK), and PH4CV + 180 mmol/L NaCl (PN). The experiment followed the method of Xian et al. [30]. The seeds were sown in a seedling pot (15 × 12 × 10 cm). The sterilized vermiculite was uniformly mixed with the corresponding solution (5 g:1 mL). The plant spacing was 10 cm. The treatment lasted for about 15 days. When the leaves of the control group grew to the three-leaf-one-heart stage, the relevant indicators were measured. For each material and each treatment, 5 plants were selected, and the average value was taken after measurement. Three replicates were set. Based on the comprehensive salt tolerance index (D value), 198 individuals were randomly selected from the F_2_ population, from which 50 salt-tolerant and 50 salt-sensitive phenotypic extremes were selected for DNA extraction and subsequent sequencing analysis. All plant samples were immediately collected after rinsing with double-distilled water, rapidly frozen in liquid nitrogen for 3–4 h, and then stored at −80 °C until further use.

### 2.2. Phenotypic Characterization

#### 2.2.1. Growth Parameter Analysis

Uniformly developed seedlings were selected for measurement of growth parameters. Root systems were carefully rinsed with distilled water to remove adhering vermiculite, followed by gentle blotting with filter paper to eliminate surface moisture. Morphological traits, including shoot length (SL), root length (RL), aboveground fresh weight (AFW), underground fresh weight (UFW), aboveground dry weight (ADW), and underground dry weight (UDW), were measured using a digital caliper and a precision electronic balance. All measurements were conducted in triplicate, and the mean values were calculated for subsequent statistical analysis.

#### 2.2.2. Physiological and Biochemical Parameter Assessment

Approximately 17 days after treatment, physiological and biochemical parameters of maize seedlings, including superoxide dismutase (SOD), peroxidase (POD), catalase (CAT), malondialdehyde (MDA), proline (Pro), soluble proteins (SP), and soluble sugars (SS), were determined following the method of Alici and Arabaci [31] and Wang et al. [32]. Among them, SOD activity was measured based on the inhibition of nitroblue tetrazolium (NBT) photochemical reduction, POD activity was assayed by monitoring the oxidation of guaiacol at 470 nm, and CAT activity was determined by measuring the disappearance of H_2_O_2_ at 240 nm. The contents of malondialdehyde (MDA), proline (Pro), soluble proteins (SP), and soluble sugars (SS) were also quantified spectrophotometrically; MDA was determined via the thiobabituric acid (TBA) reaction, Pro was measured using the ninhydrin method, SP was determined using the Coomassie Brilliant Blue G-250 dye-binding method, and SS was quantified using the anthrone method. Concurrently, ion contents—cations (K^+^, Na^+^, Ca^2+^) and anions (Cl^−^)—were quantified using inductively coupled plasma mass spectrometry (ICP-MS) [33]. Reagent blank controls were performed to correct for background interference, and test solutions were analyzed to determine the concentration of each element. For each genotype and treatment combination, three biological replicates were assayed, and the average values were used for data analysis.

### 2.3. BSA-Seq

Genomic DNA was extracted from each sample using a modified CTAB method [34], and DNA purity was assessed using NanoDrop 2000 and 8000 micro-spectrophotometers (Thermo Fisher Scientific, Waltham, MA, USA). A total of 100 samples meeting the required quality criteria were submitted to Annoroad Gene Technology Co., Ltd. (Beijing, China). for library preparation and deep sequencing. High-quality DNA samples were pooled in equimolar amounts and diluted to a final concentration of 70 ng/μL to construct two bulked DNA pools representing extreme phenotypes: the salt-tolerant pool (ST) and the salt-sensitive pool (SS). Sequencing was performed on the BGI DNBSEQ-T7 high-throughput sequencing platform (BGI, Shenzhen, China) using a paired-end strategy, with an average sequencing depth of 20×. Raw sequencing data were processed using CASAVA version 1.8.2 (Illumina, San Diego, CA, USA) [35] for base calling to generate raw reads. Subsequently, stringent quality filtering was applied to remove adapter sequences, reads containing more than 5% ambiguous bases (N), and low-quality reads, yielding high-quality clean data for downstream analysis.

Confidence intervals for the Euclidean Distance 4 (ED4) and Ridit values were calculated using Linux operating system (CentOS version 7.0) and the R version 4.2.1 (R Foundation for Statistical Computing, Vienna, Austria) [36]. Among them, ED^4^ enhances the contrast between true QTL regions and background noise by amplifying allele frequency differences between bulks, while Ridit provides a non-parametric, distribution-relative measure suitable for multi-bulk comparisons [37,38]. Integrated analysis was conducted by aligning these results with the B73 Reference Genome Version 5.0 from the Maize Genetics and Genomics Database (Maize GDB) and supplementary genomic annotations from NCBI. This enabled the identification and functional annotation of genes located within the candidate genomic intervals, providing detailed gene descriptions for further investigation.

### 2.4. Transcriptome Analysis

#### 2.4.1. Experimental Design and Sample Preparation

Samples derived from Zheng58 and PH4CV under control and salt stress conditions were used for transcriptome analysis. Total RNA was extracted using Trizol reagent (Invitrogen, Carlsbad, CA, USA) [39], and the concentration and integrity of the isolated nucleic acid were assessed using the NanoDrop 2000 spectrophotometer (Thermo Fisher Scientific, Waltham, MA, USA) and the Agilent 2100 Bioanalyzer (LabChip GX Touch) (Agilent Technologies, Santa Clara, CA, USA) [40], respectively. Following rigorous quality assessment, only RNA samples meeting the stringent criteria of a RIN ≥ 7.0 and 260/280 ratios of 1.9–2.1 proceeded to library preparation for RNA-seq analysis (Supplementary Table S1). Transcriptome sequencing of the 12 cDNA libraries was performed using the Illumina NovaSeq 6000 platform (Illumina, San Diego, CA, USA). Clean data were generated, and sequencing quality was assessed based on Q30 scores and GC content. Raw reads were aligned to the maize B73 reference genome (Version 5.0), and mapping rates were calculated. These quality control metrics ensured the reliability of the sequencing data for subsequent gene expression quantification and differential expression analyses (Supplementary Table S2).

#### 2.4.2. RNA Extraction and Library Preparation

Following sequencing, quality control was performed on the raw reads to generate high-quality clean data through the filtering of low-quality sequences and technical artifacts. The resulting clean reads were aligned to the maize reference genome B73 (Zm_B73_REFERENCE_GRAMENE_5.0) using HISAT2 version 2.2.1 [41]. After alignment, paired-end reads were assembled and quantified at the transcript level using StringTie version 2.2.0 [42].

#### 2.4.3. Differential Gene Expression Analysis

Differential gene expression analysis was conducted using the DESeq2 and EBSeq pipelines [43]. Gene expression levels were quantified and normalized using the FPKM (Fragments Per Kilobase of transcript per Million mapped reads) metric. DEGs (Differentially expressed genes) were identified based on stringent criteria: |log_2_ fold change| ≥ 1 and adjusted *p*-value < 0.05. Following the comparative analysis across all samples, bidirectional hierarchical clustering of DEGs and samples within each treatment group was performed using the pheatmap package version 1.0.12 (R package) [44] and ComplexHeatmap package version 2.14.0 (R package) [45]. Subsequently, clusterProfiler package version 4.6.0 (R package) [46] was used to conduct GO (Gene Ontology) enrichment analysis and KEGG (Kyoto Encyclopedia of Genes and Genomes) pathway analysis on the identified differentially expressed genes (DEGs).

### 2.5. Quantitative Real-Time PCR Validation

To validate the RNA-seq results, RT-qPCR was performed on 32 candidate genes (Supplementary Table S3) using gene-specific primers designed with Primer-BLAST (NCBI, https://www.ncbi.nlm.nih.gov/tools/primer-blast/, 13 October 2025) [47]. Total RNA was reverse transcribed into cDNA using the RNA Simple Total RNA Kit (Tiangen, Shanghai, China). The actin gene was employed as an internal reference gene [48], and the relative expression levels of target genes were calculated using the 2^−ΔΔCT^ method [49]. The results were used to confirm the reliability and accuracy of the transcriptome data.

### 2.6. The Expression Specificity of Candidate Gene in Different Tissues

To further investigate the tissue-specific expression of the *ZmACC2* gene in maize plants [50,51], we used RT-qPCR technology to examine the expression pattern of the *ZmACC2* gene in different tissues of maize. The experiment included three tissues (root, stem and leaf), applied to six varieties of maize (PH4CV, Zheng58, N192, Qi319, Mo17 and B73), and two treatment conditions—distilled water (CK) and 180 mmol/L NaCl (N), resulting in a total of 36 treatment groups. The group codes are composed as follows: variety abbreviation (P, Z, N, Q, M, or B) + treatment (CK or N) + tissue (R, S, or L). Specifically, the 36 groups are: PCK-R, PCK-S, PCK-L, ZCK-R, ZCK-S, ZCK-L, NCK-R, NCK-S, NCK-L, QCK-R, QCK-S, QCK-L, MCK-R, MCK-S, MCK-L, BCK-R, BCK-S, BCK-L, PN-R, PN-S, PN-L, ZN-R, ZN-S, ZN-L, NN-R, NN-S, NN-L, QN-R, QN-S, QN-L, MN-R, MN-S, MN-L, BN-R, BN-S, BN-L.

### 2.7. Statistical Analysis

Experimental data were initially processed using Microsoft Excel 2019. Statistical analyses, including paired-sample *t*-tests and correlation analyses, were conducted using IBM SPSS Statistics 27.0 and Origin 2022. Data are presented as mean ± standard error (SE) derived from at least three independent biological replicates. Significance levels were set at *p* < 0.05 unless otherwise specified.

## 3. Results

### 3.1. Salt Tolerance Test of Zheng58 and PH4CV

#### 3.1.1. Growth Index Under Salt Stress

**Figure 1:**
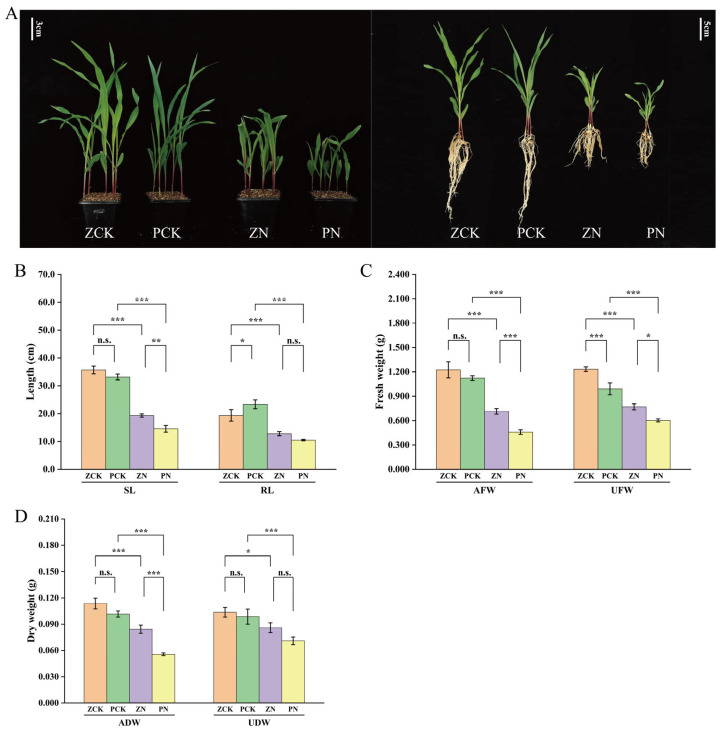
Growth indexes of seedlings under different treatments. (**A**) Effects of salt stress on maize seedlings, from left to right as follows: Zheng58 + distilled water (ZCK), Zheng58 + NaCl (ZN), PH4CV + distilled water (PCK), and PH4CV + NaCl (PN). (**B**) SL and RL. (**C**) AFW and UFW. (**D**) ADW and UDW. Significance levels are indicated as: n.s., no significance; *, *p* < 0.05; **, *p* < 0.01; ***, *p* < 0.001.

Analysis of growth parameters in Zheng58 and PH4CV revealed that salt stress significantly inhibited seedling growth compared to the control (distilled water treatment). Under the NaCl treatment, SL, RL, AFW, UFW, ADW and UDW all exhibited a marked decline in both genotypes. Specifically, in the salt-sensitive variety PH4CV, these indices decreased by 56.08%, 55.06%, 59.18%, 39.19%, 45.25%, 28.04%, respectively (note: values may correspond to paired control vs. stress comparisons across the six traits, with potential duplication or formatting artifact in reporting). In contrast, the salt-tolerant Zheng58 showed smaller reductions of 45.85%, 33.91%, 41.69%, 37.67%, 25.81%, 17.04%, respectively, indicating a stronger capacity to maintain growth under stress. Statistical analysis revealed significant (*p* < 0.01) or highly significant (*p* < 0.001) differences in most growth traits between the two varieties under salt stress conditions. These results demonstrate that salt stress exerts a strong inhibitory effect on maize seedling growth, and Zheng58 exhibits superior salt tolerance compared to PH4CV, as reflected by its relatively higher performance across all morphological and biomass-related indices (Figure 1A–D).

#### 3.1.2. Physiological and Biochemical Indicators Under Salt Stress

**Figure 2:**
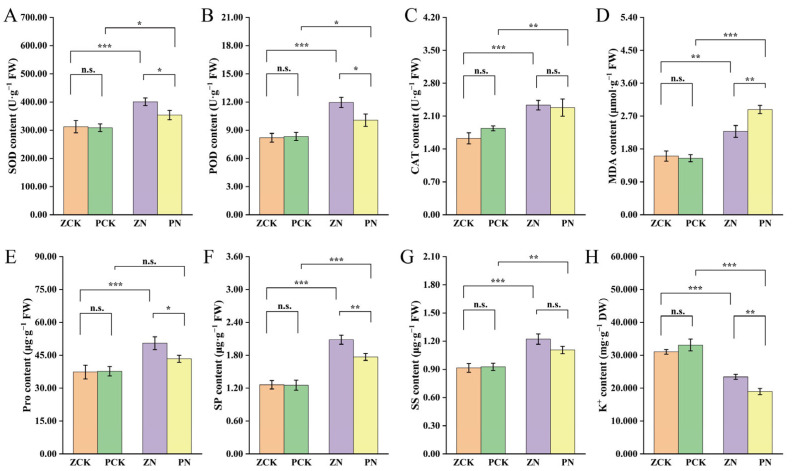

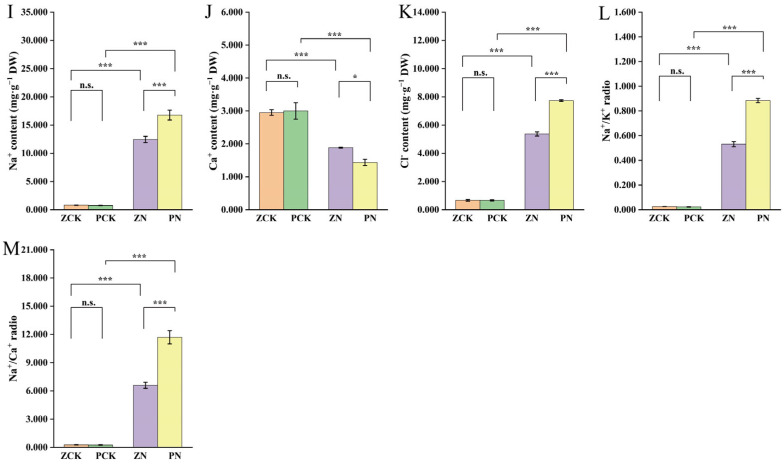
Physiological and biochemical indices of seedlings under different treatments. (**A**) SOD content. (**B**) POD content. (**C**) CAT content. (**D**) MDA content. (**E**) Pro content. (**F**) SP content. (**G**) SS content. (**H**) K^+^ content. (**I**) Na^+^ content. (**J**) Ca^2+^ content. (**K**) Cl^−^ content. (**L**) Na^+^/K^+^ ratio. (**M**) Na^+^/Ca^2+^ ratio. Significance levels are indicated as: n.s., no significance; *, *p* < 0.05; **, *p* < 0.01; ***, *p* < 0.001.

Analysis of physiological and biochemical parameters in Zheng58 and PH4CV revealed that, under the NaCl stress, all measured indicators—SOD, POD, CAT, MDA, Pro, SP, and SS—exhibited varying degrees of increase compared to their respective controls (Figure 2A–G). However, the magnitude of change differed significantly between the two genotypes, reflecting their contrasting salt tolerance capacities. MDA, a key indicator of membrane lipid peroxidation and oxidative damage, increased by 86.08% in the sensitive variety PH4CV and by only 42.02% in the tolerant variety Zheng58 relative to their controls. This substantial accumulation in PH4CV suggests greater cellular damage under salt stress. In contrast, antioxidant enzyme activities showed a more favorable response in Zheng58. SOD, POD, and CAT are crucial components of the plant’s antioxidative defense system, responsible for scavenging ROS. Under salt stress, PH4CV exhibited increases of 14.64% (SOD), 20.92% (POD), and 23.91% (CAT), while Zheng58 showed significantly higher enhancements of 28.31%, 45.72%, and 43.44%, respectively. This indicates that Zheng58 possesses a more robust antioxidant capacity, effectively mitigating oxidative stress. Osmotic substances such as Pro, SP, and SS play vital roles in osmotic adjustment, protein stabilization, and membrane protection. Their accumulation helps maintain cellular turgor and protects macromolecules under stress conditions. Under salt stress, PH4CV showed increases of 15.10% (Pro), 41.18% (SP), and 19.33% (SS) compared to the control. In comparison, Zheng58 exhibited much greater accumulation—35.26% (Pro), 65.04% (SP), and 33.53% (SS)—demonstrating a superior ability to adjust osmotically and stabilize cellular structures.

Salt stress significantly altered ion homeostasis in both maize varieties, leading to a general increase in Na^+^ and Cl^−^ concentrations and a concurrent decrease in K^+^ and Ca^2+^ levels compared to the control (Figure 2H–M). These shifts resulted in a substantial rise in the Na^+^/K^+^ and Na^+^/Ca^2+^ ratios, which are critical indicators of salt-induced ionic imbalance and toxicity. Under control conditions (distilled water), no significant differences in ion content or ratios were observed between Zheng58 and PH4CV. However, upon exposure to NaCl, marked genotypic differences emerged. Zheng58 maintained significantly higher levels of essential cations: its K^+^ content was 1.24 times and Ca^2+^ content 1.31 times higher than those in PH4CV under salt stress, reflecting a superior ability to retain these vital nutrients. In contrast, PH4CV exhibited a much greater accumulation of toxic ions. Na^+^ content increased by 2100.40% and Cl^−^ by 1064.43% relative to its control, whereas Zheng58 showed comparatively lower increases of 1443.40% (Na^+^) and 701.58% (Cl^−^). More strikingly, the Na^+^/K^+^ and Na^+^/Ca^2+^ ratios—key determinants of cellular ionic balance—increased by 3731.44% and 4479.53%, respectively, in PH4CV compared to its control. In Zheng58, the same ratios increased by 1944.11% and 2313.91%, respectively, indicating a significantly better capacity to restrict Na^+^ influx and preserve K^+^ and Ca^2+^ homeostasis. These findings demonstrate that under salt stress, maize seedlings respond by modulating the uptake, transport, and compartmentalization of key ions. The salt-tolerant variety Zheng58 exhibits enhanced ion selectivity and regulation, minimizing Na^+^ and Cl^−^ accumulation while preserving higher levels of K^+^ and Ca^2+^. This effective ion balance helps maintain membrane stability, enzyme activity, and overall cellular function, thereby contributing to its superior salt tolerance. In contrast, the excessive Na^+^/K^+^ and Na^+^/Ca^2+^ ratios in PH4CV suggest severe disruption of ion homeostasis, leading to greater physiological damage and reduced stress resilience.

### 3.2. Salt Tolerance Candidate Gene Identification Based on BSA-Seq

**Figure 3:**
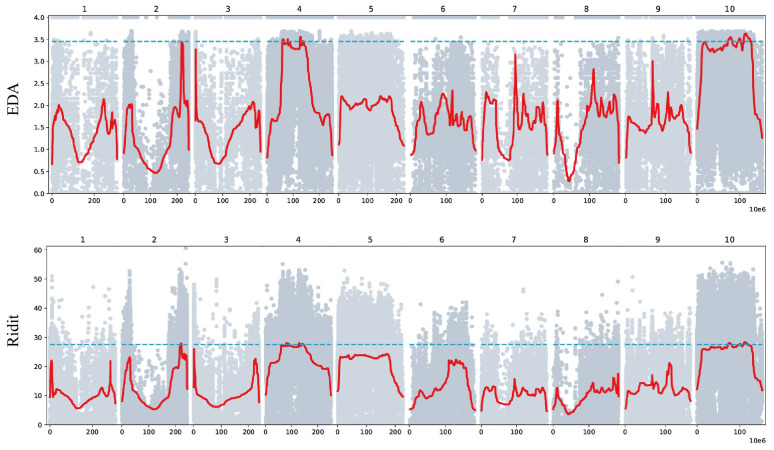
Manhattan diagram of maize salt tolerance traits based on BSA. The abscissa is the chromosome name, the blue dotted line represents the significant correlation threshold, and the red line represents the fitting line after the window slides. Among them, grey dots represent SNP markers below the significance threshold.

The candidate genomic intervals associated with salt tolerance were identified using two complementary analytical methods: ED4 and Ridit. According to the predefined significance thresholds, the candidate regions detected by the ED4 algorithm were found to be largely nested within the highly significant intervals identified by the Ridit method. The overlapping regions between these two approaches were considered high-confidence candidate intervals linked to salt tolerance. Ultimately, six such consensus regions were confirmed (Figure 3), located on chromosomes 4 and 10. The combined length of these regions spanned 28.60 Mb, with individual sizes of 2.68 Mb, 3.16 Mb, 1.89 Mb, 7.95 Mb, 3.25 Mb, and 9.67 Mb, respectively (Supplementary Table S4).

Following the delineation of these confidence intervals, gene annotation information within each region was retrieved from the Maize GDB (https://www.maizegdb.org/ (accessed on 13 October 2025)) to identify potential candidate genes. A total of 391 annotated genes were located within the six combined intervals (Supplementary Table S5). Subsequent functional annotation and screening—based on gene function, biological processes, and relevance to abiotic stress responses—were performed using integrated data from Maize GDB and the NCBI database (https://www.ncbi.nlm.nih.gov/ (accessed on 17 October 2025)). After filtering for genes potentially involved in ion transport, osmotic regulation, signal transduction, and stress-responsive pathways, a subset of 151 putative salt tolerance-related candidate genes was selected for further analysis (Supplementary Table S6). These genes represent promising targets for dissecting the genetic basis of salt tolerance in maize.

### 3.3. Transcriptome Analysis of the Seedling Stage of the Two Varieties Under Salt Stress

#### 3.3.1. Differential Gene Expression Analysis

As shown in Supplementary Figure S1A, under salt stress, a total of 2435 DEGs were detected in Zheng58 compared to its control, comprising 1009 up-regulated and 1426 down-regulated genes, representing 41.44% and 58.56% of the total DEGs, respectively. In contrast, the PH4CV exhibited a much broader transcriptional response, with 5788 DEGs identified, including 2652 up-regulated (45.82%) and 3136 down-regulated (54.18%). Venn diagram analysis across the comparison groups—PCK vs. ZCK, PN vs. ZN, ZCK vs. ZN, and PCK vs. PN—revealed 187 common DEGs shared between the two genotypes under stress (Supplementary Figure S1B–D). Among these, 19 genes were consistently up-regulated across all four comparison groups, suggesting their potential core role in the general response to salt stress. Conversely, 20 genes were uniformly down-regulated in all comparisons, possibly indicating conserved repression of specific biological processes under stress conditions. Additionally, differentially expressed genes (DEGs) were categorized into overlapping and uniquely expressed sets across the four treatment comparisons—PCK vs. ZCK, PN vs. ZN, ZCK vs. ZN, and PCK vs. PN. A total of 809, 1893, 522, and 1256 uniquely expressed DEGs were identified in each respective comparison group. This highlights the distinct regulatory strategies employed by the two varieties. The commonly and specifically expressed DEGs provide valuable candidates for further functional studies on salt tolerance mechanisms in maize.

**Figure 4:**
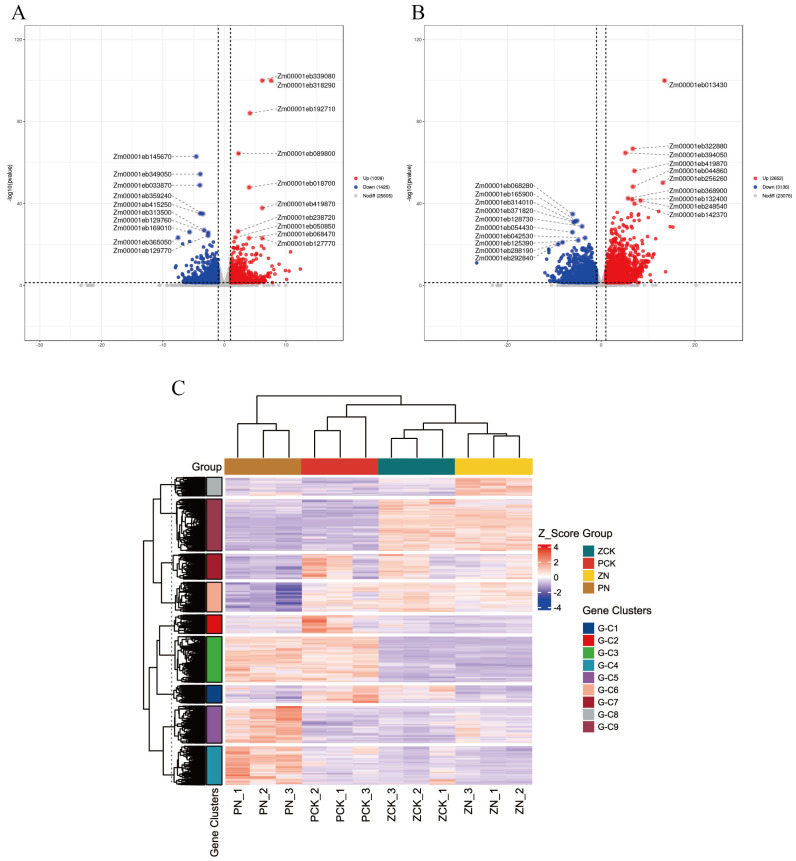
(**A**,**B**) Volcano map based on differential expression results: (**A**) ZCK vs. ZN and (**B**) PCK vs. PN. (**C**) The expression levels of the same gene in different samples and the expression patterns of different genes in the same sample were clustered. Among them, the dashed line represents the significance threshold.

Based on the differential expression analysis, volcano plots were generated to visualize the distribution of gene expression changes between control and salt-stressed samples for both maize varieties (Figure 4A,B). In the ZCK vs. ZN comparison, several genes exhibited significant up-regulation, including *Zm00001eb339080*, *Zm00001eb318290*, *Zm00001eb192710*, *Zm00001eb089800*, and *Zm00001eb018700*. These may represent key stress-responsive candidates in the tolerant genotype. Down-regulated genes in Zheng58 under salt stress included *Zm00001eb145670*, *Zm00001eb349050*, *Zm00001eb033870*, *Zm00001eb359240* and *Zm00001eb415250* suggesting potential repression of growth- or development-related processes under stress. In the PCK vs. PN comparison, up-regulated genes included *Zm00001eb013430*, *Zm00001eb322880*, *Zm00001eb394050*, *Zm00001eb419870*, and *Zm00001eb044860*, while down-regulated genes encompassed *Zm00001eb068280*, *Zm00001eb165900*, *Zm00001eb314010*, *Zm00001eb371820*, and *Zm00001eb128730* (Supplementary Table S7).

To further explore expression patterns across all samples, hierarchical clustering analysis was performed based on gene expression levels (Figure 4C). The analysis clustered both genes with similar expression profiles across samples and samples with similar gene expression patterns. The Euclidean distance was used to measure dissimilarity, and the Complete Linkage (farthest neighbor) method was applied for cluster merging. The clustering results revealed distinct co-expression patterns: Genes in clusters G-C6 and G-C9 showed high expression levels in both ZCK and ZN, suggesting constitutive or stress-enhanced activation in the tolerant variety. Clusters G-C8, G-C4, and G-C5 exhibited elevated expression specifically in ZN or PN, indicating stress-induced activation unique to each genotype. Cluster G-C3 displayed high expression in both PCK and PN, implying a baseline expression bias in PH4CV that is maintained regardless of stress condition.

#### 3.3.2. GO Function Enrichment Analysis

**Figure 5:**
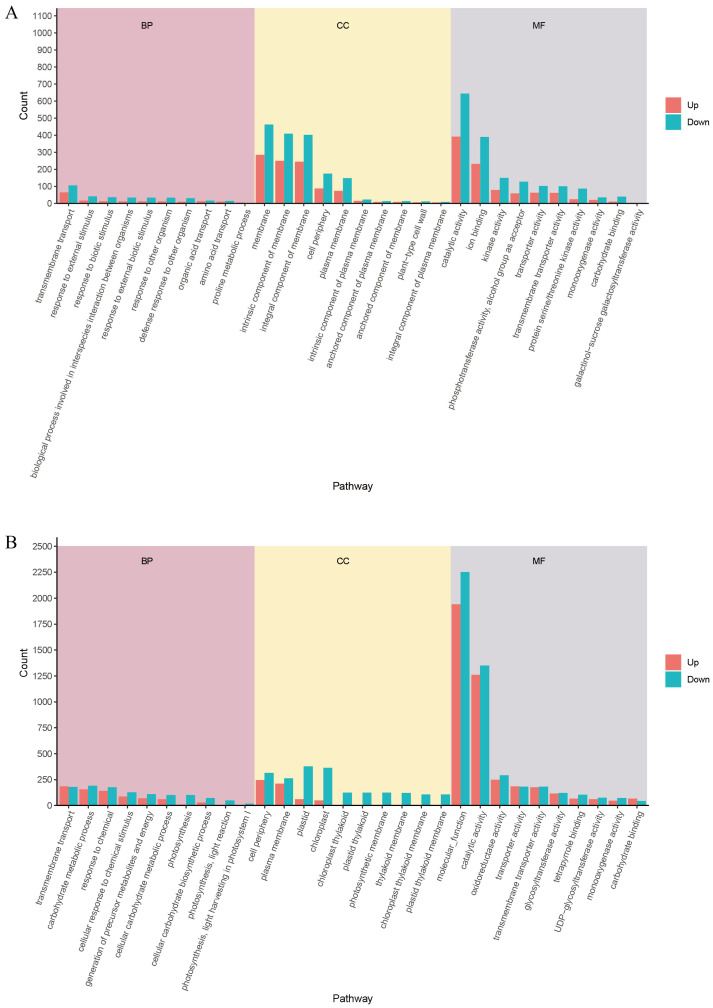

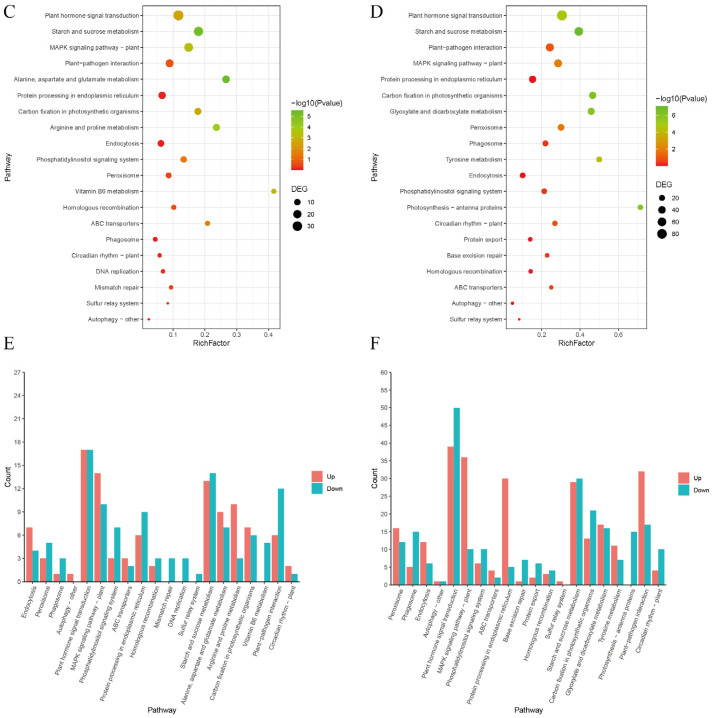
Comparative functional enrichment analysis of DEGs in Zheng58 and PH4CV under control and salt stress conditions. (**A**,**B**) GO enrichment analysis of DEGs. (**C**,**D**) Scatter plots of KEGG pathway enrichment results. Dot size reflects the number of DEGs annotated to each pathway (larger dots, more DEGs). The color gradient from red to green represents the statistical significance of enrichment, with red indicating high significance (small *p*-value) and green indicating low significance (large *p*-value). (**E**,**F**) KEGG enrichment analysis of up-regulated and down-regulated DEGs. Note: (**A**,**C**,**E**) correspond to the ZCK vs. ZN comparison (Zheng58), while (**B**,**D**,**F**) correspond to the PCK vs. PN comparison (PH4CV).

To elucidate the biological functions and processes associated with the DEGs in response to salt stress, GO functional enrichment analysis was performed for both Zheng58 and PH4CV. The analysis categorized significantly enriched GO terms into three main domains: Biological Process (BP), Cellular Component (CC), and Molecular Function (MF), with up- and down-regulated genes analyzed separately (Figure 5A,B). Compared with the control group, under salt stress treatment, the terms of Zheng58 and PH4CV that were significantly upregulated in BP, CC and MF were 220/12/127, 163/36/69, and the terms that were significantly downregulated were 239/12/131, 172/41/68. In Zheng58, the most significantly enriched BP included transmembrane transport, response to external stimulus, response to biotic stimulus, biological processes involved in interspecies interaction, and response to other organisms. The enriched CC were predominantly associated with membrane structures, including membrane, intrinsic component of membrane, integral component of membrane, cell periphery, and plasma membrane. For MF, key enriched terms were catalytic activity, ion binding, kinase activity, phosphotransferase activity, and transporter activity. The GO enrichment profile in PH4CV showed largely similar functional categories, suggesting conservation in the types of biological processes affected by salt stress. However, Zheng58 exhibited fewer DEGs within the same enriched terms, indicating a more targeted and potentially efficient transcriptional response compared to the broader and possibly less specific activation in PH4CV. Overall, the DEGs identified in this study are primarily involved in transmembrane transport and are localized to the cell periphery and plasma membrane. Key molecular functions include catalytic activity, transporter activity, transmembrane transporter activity, monooxygenase activity, and carbohydrate binding.

#### 3.3.3. KEGG Pathway Enrichment Analysis

To gain deeper insights into the biological roles and metabolic pathways associated with DEGs under salt stress, KEGG pathway enrichment analysis was conducted for both Zheng58 and PH4CV. The results revealed distinct patterns of pathway activation and suppression between the two genotypes (Figure 5C,D). In Zheng58, 22 out of 111 annotated pathways were significantly enriched under salt stress (Supplementary Table S8). Key enriched pathways included: Plant hormone signal transduction, Starch and sucrose metabolism, MAPK signaling pathway–plant, Plant–pathogen interaction, Alanine, aspartate and glutamate metabolism. In contrast, PH4CV exhibited significant enrichment in 36 out of 124 annotated pathways (Supplementary Table S9). While several pathways overlapped with those in Zheng58—such as Plant hormone signal transduction, Starch and sucrose metabolism, Plant–pathogen interaction, MAPK signaling pathway–plant. Additionally, PH4CV showed enrichment in unique stress-related pathways such as: Glyoxylate and dicarboxylate metabolism, Tyrosine metabolism, Photosynthesis—antenna proteins. Interestingly, several pathways were uniquely enriched in Zheng58, including: Alanine, aspartate and glutamate metabolism, Arginine and proline metabolism, Vitamin B6 metabolism.

Further examination of gene expression trends within these pathways (Figure 5E,F) revealed genotype-specific regulatory strategies: In Zheng58, genes involved in Plant hormone signal transduction, MAPK signaling pathway-plant, Starch and sucrose metabolism, and Arginine and proline metabolism were predominantly up-regulated, indicating active stress signaling and osmotic regulation. Conversely, genes in Plant-pathogen interaction, Protein processing in endoplasmic reticulum, and Phosphatidylinositol signaling system were down-regulated, possibly reflecting resource reallocation away from secondary metabolism and certain lipid signaling pathways. In PH4CV, up-regulation was observed in Plant-pathogen interaction, MAPK signaling pathway–plant, Protein processing in endoplasmic reticulum, and Peroxisome. However, significant down-regulation occurred in key processes such as Plant hormone signal transduction, Carbon fixation in photosynthetic organisms, Phagosome, and Photosynthesis—antenna proteins.

### 3.4. Identification of Salt Tolerance Candidate Genes

To refine the list of potential salt tolerance genes and identify high-confidence candidates, an integrated analysis was performed by combining results from BSA-seq and RNA-seq. This multi-omics approach enabled the prioritization of candidate genes located within significant QTL intervals that also exhibited differential expression under salt stress. From the initial set of 151 candidate genes identified within the six confidence intervals through functional annotation, 129 genes were found to be plausibly associated with salt stress responses based on their genomic location, expression patterns, and functional relevance. Among these, 69 genes showed detectable and reliable expression levels across the RNA-seq datasets, confirming their transcriptional activity under both control and salt-stressed conditions. Based on gene functional annotation analysis (Supplementary Table S10), a total of 9 and 23 high-confidence candidate genes were identified within the 5.93 Mb interval on chromosome 4 and the 4.58 Mb interval on chromosome 10, respectively (Table 1). These genes are primarily involved in three key biological processes associated with salt stress response in maize: Ion transport and homeostasis: including K16055 (K^+^ transport), K14652 (Na^+^/H^+^ antiporter), K14808 (vacuolar H^+^-ATPase), and K10251 (plasma membrane H^+^-ATPase); Antioxidant defense and ROS scavenging: K01922, K02335, K00799, K00111, K11262, K16296, K06949, K20179, K13095, and K21596; Nitrogen metabolism and biosynthesis of osmotic regulatory substances: K00658 (glutamine synthetase) and K03696 (arginine decarboxylase, involved in polyamine synthesis).

### 3.5. RT-qPCR (Quantitative Real-Time PCR) Validation of the Candidate Genes

In order to further confirm the reliability of the transcriptome sequencing results and identify candidate genes that play a role in the salt tolerance pathway, we conducted RT-qPCR comparison and verification (Supplementary Figure S2) for 32 DEGs (Supplementary Table S11). The RNA-seq results were consistent with the RT-qPCR results, indicating the reliability of the sequencing results. To prioritize candidate genes from the 32 identified candidates, we first examined their expression trends under salt stress. *Zm00001eb419400* (*ZmACC2*, chr10:100241710-100263391) exhibited consistent and significant upregulation, with a greater fold change compared to other candidates. Next, we focused on genes annotated in plant hormone signal transduction and MAPK signaling pathways, as these are well-established regulators of plant stress responses. Among these, *ZmACC2*, which encodes an ACC synthase involved in ethylene biosynthesis, was prioritized due to its functional relevance in hormone signaling and its potential crosstalk with MAPK cascades under salt stress. Based on these explicit selection criteria, *ZmACC2* was identified as the core candidate gene for further analysis (Supplementary Table S12).

### 3.6. Analysis of Tissue-Specific Differential Expression of the ZmACC2 Gene

**Figure 6:**
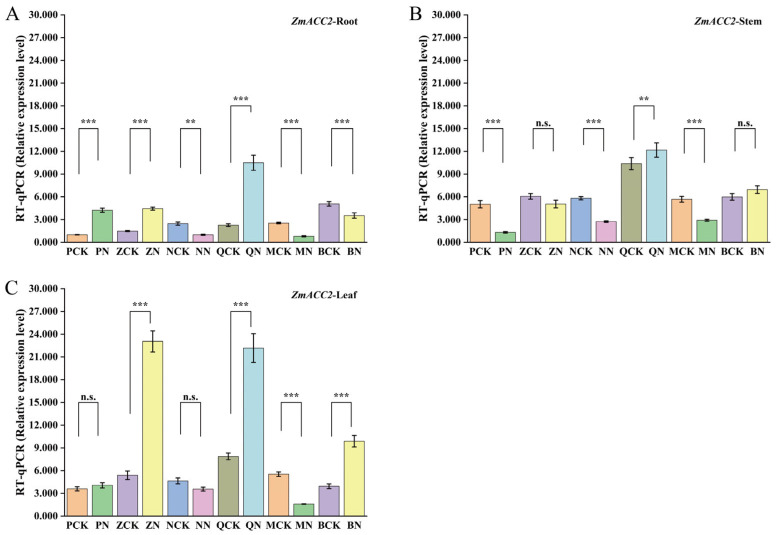
Analysis of the relative expression of *ZmACC2* in different tissues in different varieties of maize. Note: The experiment included three tissues, applied to six varieties of maize, and two treatment conditions, resulting in a total of 36 treatment groups: PCK-R, PCK-S, PCK-L, ZCK-R, ZCK-S, ZCK-L, NCK-R, NCK-S, NCK-L, QCK-R, QCK-S, QCK-L, MCK-R, MCK-S, MCK-L, BCK-R, BCK-S, BCK-L, PN-R, PN-S, PN-L, ZN-R, ZN-S, ZN-L, NN-R, NN-S, NN-L, QN-R, QN-S, QN-L, MN-R, MN-S, MN-L, BN-R, BN-S, BN-L. Among them, (**A**) Root; (**B**) Stem; (**C**) Leaf. Significance levels are indicated as: n.s., no significance; **, *p* < 0.01; ***, *p* < 0.001.

As shown in Figure 6, there are significant differences in the expression pattern of the *ZmACC2* gene in different tissues of maize. If the expression level of the *ZmACC2* gene in roots is converted to 1, the expression level of *ZmACC2* in maize leaves is the highest, and the expression levels in the leaves of Zheng58 and Qi319 are significantly higher than those in the roots. The results indicate that the higher expression of *ZmACC2* in leaves compared to roots and stems under salt stress suggests a potential leaf-specific role (Supplementary Tables S13 and S14).

## 4. Discussion

Salinization is a major abiotic stress that adversely affects maize growth and development, often leading to significant yield losses [52,53,54]. Beyond growth inhibition, salt stress disrupts key physiological processes, including the antioxidant system and osmolyte metabolism. In the present study, Zheng58 and PH4CV showed comparable antioxidant enzyme activities under normal conditions. However, under salt stress, Zheng58 exhibited significantly higher activities of SOD, POD, and CAT—1.13-, 1.19-, and 1.02-fold higher than PH4CV, respectively—suggesting a more robust activation of its antioxidant defense. This aligns with observations in other crops, where coordinated upregulation of these enzymes enhances salt tolerance by efficiently scavenging ROS and preventing oxidative damage [55]. Concurrently, Zheng58 accumulated higher levels of osmotic regulatory substances: Pro was 1.16-fold higher than in PH4CV, while SP and SS were elevated by 1.18- and 1.10-fold, respectively. Pro not only contributes to osmotic adjustment but also stabilizes proteins and membranes under salt-induced water stress [56], reinforcing Zheng58’s superior osmoregulatory capacity. Consistent with the heightened oxidative stress, MDA content markedly increased in leaf tissues under salt treatment, indicating severe lipid peroxidation and membrane damage—a common consequence of ROS accumulation in photosynthetically active tissues [57].

During the maize seedling stage, salt stress induces ionic imbalance by altering the concentrations of key ions such as Na^+^, K^+^, Ca^2+^, and Cl^−^, thereby disrupting normal physiological processes [58]. Excessive Na^+^ accumulation not only disturbs intracellular ion homeostasis but also interferes with the uptake and translocation of essential cations like K^+^ and Ca^2+^, compromising membrane stability and enzyme activity [59,60]. Potassium (K^+^) is a critical cofactor for numerous enzymes and plays central roles in osmotic regulation, stomatal control, and antioxidant defense. Under salt stress, reduced K^+^ uptake and increased efflux—as observed in asparagus—lead to lower intracellular K^+^, higher Na^+^, and an elevated Na^+^/K^+^ ratio, a key indicator of ionic stress [54]. Calcium (Ca^2+^) acts as a vital secondary messenger in stress signaling, contributing to membrane stabilization and regulation of ion channels, thereby enhancing selective permeability and salt tolerance [61]. Chloride (Cl^−^), while necessary for charge balance and osmotic potential, becomes phytotoxic at high levels, impairing metabolism and growth in maize [62]. In this study, Zheng58 accumulated higher levels of Na^+^ and Cl^−^ than PH4CV under salt stress, which may reflect more efficient sequestration of these ions into vacuoles or less sensitive tissues, minimizing their cytotoxic effects in metabolically active leaf cells. Concurrently, Zheng58 maintained relatively higher K^+^ and Ca^2+^ concentrations, suggesting a superior ability to preserve ion homeostasis. Given that leaves are highly sensitive to ionic disturbances, this balanced ion management likely contributes to Zheng58’s enhanced salt tolerance during the seedling stage. These findings indicate that genotypic differences in salt tolerance are closely linked to strategies for ion uptake, transport, and compartmentalization. While variation in energy allocation, protective metabolite synthesis, and ion transport efficiencyunderlie these differences, the genetic and molecular mechanisms remain incompletely understood. Systematic identification and functional characterization of salt-tolerance genes and their regulatory networks are therefore essential to elucidate these processes and provide a foundation for breeding salt-resilient maize varieties.

Plant hormone signal transduction and MAPK signaling pathways are central to plant responses to abiotic stresses, coordinating physiological adjustments such as osmotic regulation, ion homeostasis, and antioxidant defense (Supplementary Figure S3). The MAPK signaling cascade transduces stress signals into downstream responses, including hormone signal transduction and gene expression [63,64]. Multiple MAPK pathway genes were differentially expressed under salt stress in our transcriptome data, consistent with their known roles in salt tolerance, particularly MPK3/6 (Mitogen-Activated Protein Kinase 3/6), which directly phosphorylate and stabilize ACC synthases (ACSs), thereby modulating ethylene production, suggesting coordination between MAPK and ethylene pathways in salt stress response [65,66,67]. Ethylene, a gaseous phytohormone, regulates diverse processes including growth, development, and stress responses [68,69]. Given its central role in abiotic stress adaptation, modulating ethylene biosynthesis or signaling offers promising avenues for crop improvement. 1-Aminocyclopropane-1-carboxylate oxidase (ACO) catalyzes the final step of ethylene biosynthesis and is critical for stress-induced ethylene production [70]. In peanut, overexpression of *AhACO1* and *AhACO2* from a salt-tolerant mutant enhanced growth, photosynthetic performance, and ethylene emission under salt stress, indicating a positive role of ACO in tolerance [71]. Similarly, ACOh4 in another species was shown to improve K^+^/Na^+^ homeostasis via Na^+^/H^+^ antiport activity, linking ACO-mediated ethylene production to ion regulation [72]. Beyond biosynthesis, ethylene signaling is equally important. The transcription factor EIN3 acts as a central hub in this pathway. Overexpression of GhEIN3 in transgenic plants enhanced salt tolerance by reducing H_2_O_2_ and MDA accumulation, boosting antioxidant enzyme activities (SOD, POD), and upregulating ROS- and ABA-related genes [73]. Likewise, rice OSERF19, an ethylene-responsive factor, conferred improved salt tolerance when overexpressed despite being downregulated under stress, underscoring the functional importance of ethylene-responsive elements [74]. In our study, the ethylene pathway was differentially regulated between PH4CV (salt-sensitive) and Zheng58 (salt-tolerant). Under salt stress, PH4CV showed upregulation of EIN3 (*Zm00001eb244830*, Ethylene Insensitive 3-like 5 protein) and ERF1/2 (*Zm00001eb307550*, Ethylene Response Factor). In contrast, Zheng58 exhibited downregulation of EBF1/2 (*Zm00001eb276590*, Ethylene Response Factor)—a negative regulator that targets EIN3 for degradation—alongside upregulation of EIN3 (*Zm00001eb331080*, protein Ethylene-insensitive 3-like 2), suggesting enhanced EIN3 protein stability in the tolerant genotype. Based on these findings and the established roles of ACO and EIN3 in stress adaptation, we hypothesize that *ZmACC2* (1-aminocyclopropane-1-carboxylate synthase, ACS) contributes to salt tolerance in Zheng58 by (1) promoting degradation of EBF1/2, thereby stabilizing EIN3 and activating downstream stress-responsive genes, and (2) potentially regulating ion homeostasis—particularly Na^+^, K^+^, and Ca^2+^ balance—to enhance cellular stability under salinity. Under NaCl stress, ACC significantly enhanced Na^+^ extrusion, mitigated K^+^ loss, and elevated both H_2_O_2_ accumulation and intracellular membrane Ca^2+^ levels induced by salt. Based on these findings, Lang et al. proposed that ethylene, triggered by extracellular ATP (eATP) as a novel upstream signaling component, subsequently activates and potentiates the H_2_O_2_ and Ca^2+^ signaling pathways, thereby contributing to the maintenance of K^+^/Na^+^ homeostasis under saline conditions [75]. Zhang et al. demonstrated that under 150 mmol/L NaCl stress, transgenic lines overexpressing *TaACCD* exhibited enhanced salt tolerance and faster growth than WT plants. These lines showed reduced ROS levels and electrolyte leakage, along with increased POD and SOD activities. These findings suggest that heterologous expression of *TaACCD* represents a promising strategy for enhancing plant salt tolerance [76]. At the same time, in our study, the calcium and ROS signaling components were differentially regulated between PH4CV (salt-sensitive) and Zheng58 (salt-tolerant) under salt stress. In PH4CV, CaM4 (*Zm00001eb333100*, calmodulin; *Zm00001eb352340*, probable calcium-binding protein CML9) and RbohD (respiratory burst oxidase homolog D) showed inconsistent regulation—some RbohD copies were upregulated (*Zm00001eb197890*, respiratory burst oxidase-like protein D), others downregulated (*Zm00001eb341260*, respiratory burst oxidase homolog protein B)—suggesting a dysregulated ROS–Ca^2+^ signaling network. By contrast, Zheng58 exhibited consistent downregulation of multiple CaM4 genes (*Zm00001eb279150*, EF hand family protein; *Zm00001eb196020*, calmodulin-related protein) and upregulation of a specific RbohD isoform (*Zm00001eb131620*), indicative of a more coordinated and potentially protective ROS response.

**Figure 7:**
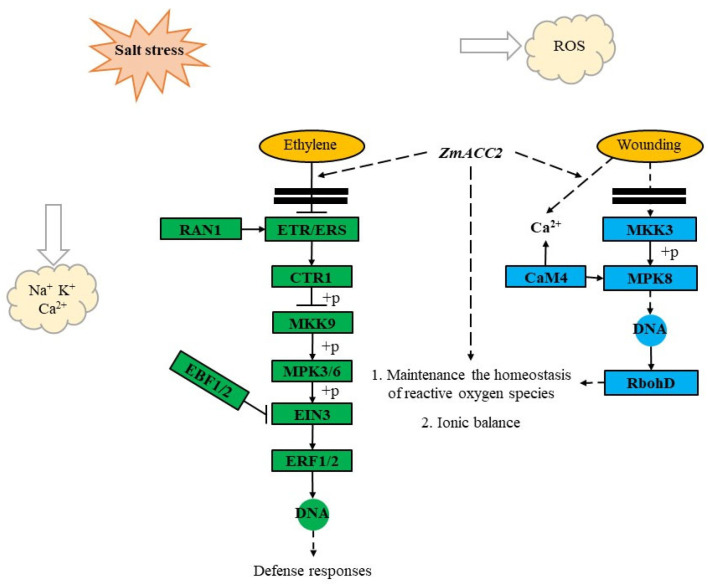
Schematic representation of hormone signaling and MAPK cascades involved in salt stress response mechanisms. The diagram illustrates key signaling pathways, with distinct color-coded nodes representing specific stress response components: The dark green frame represents the ETH signal node, and the light blue frame represents the defense response node. Schematic representation of Plant hormone signal transduction and the MAPK signaling pathway involved in salt stress response mechanisms. Among them, arrows indicate putative (speculated) regulatory interactions.

Based on these observations and the established roles of calcium signaling in stress adaptation, we propose that *ZmACC2* contributes to salt tolerance by modulating ion homeostasis via CaM4-mediated calcium signaling, while *ZmACC2* may enhance cellular protection by fine-tuning H_2_O_2_ levels and ionic balance. Together, these pathways likely act in concert to orchestrate the salt stress response in maize (Figure 7). Although our results suggest that *ZmACC2* is closely associated with salt tolerance in maize, current evidence remains preliminary. Therefore, further functional validation—including transgenic analysis, gene editing, or allelic complementation assays—will be performed to confirm the role of *ZmACC2* in salt tolerance, and to elucidate its precise molecular function and regulatory mechanism under salt stress.

## 5. Conclusions

In conclusion, this study systematically characterized the salt tolerance phenotypes and underlying genetic mechanisms of the maize varieties Zheng58 and PH4CV. By integrating BSA-seq with transcriptomic profiling, one candidate gene that may be involved in salt tolerance during the seedling stage—*ZmACC2*—was identified. We propose that under salt stress conditions, this candidate gene plays a pivotal role in maintaining cellular ion homeostasis and regulating ROS metabolism, thereby contributing to plant stress resilience and normal growth and development. Specifically, the *ZmACC2* gene maymodulate the dynamic equilibrium of key ions such as Na^+^, K^+^, and Ca^2+^ across cellular compartments. This regulatory function is potentially mediated through the modulation of central signaling components in stress-responsive pathways, including EIN3, ERF1/2, RbohD and CaM4. By fine-tuning the expression of the gene, the candidate loci may promote Na^+^ efflux or vacuolar sequestration while sustaining efficient K^+^ uptake, thus preserving optimal cytosolic ion ratios. Concurrently, the gene is implicated in the regulation of antioxidant defense systems, likely through influencing the expression ofgenes involved in ROS scavenging. This activity contributes to the effective suppression of ROS accumulation, thereby mitigating membrane lipid peroxidation and reducing oxidative damage. Through these coordinated and synergistic mechanisms—encompassing ion homeostasis, hormonal signaling, and redox balance—the identified gene enhances maize adaptability to saline environments. This enables significant improvement in salt tolerance without compromising essential physiological functions, highlighting their potential as key targets for molecular breeding and genetic engineering strategies aimed at developing salt-resilient crops. Among the candidates, *ZmACC2* emerges as particularly promising for future investigation. Both integrate core stress-response modules—ETH signaling, ion homeostasis, and ROS scavenging—and align with conserved mechanisms shown in other crops to enhance tolerance without yield loss. Prioritizing their functional validation through transgenic or gene-editing approaches, and dissecting their interactions with key signaling components (e.g., CaM4, ERF1/2, EIN3), will be critical to translate these findings into effective breeding strategies for salt-resilient maize.

## Figures and Tables

**Table 1:** Gene annotation of 32 candidate genes.

Gene	Chromosome	Gene Annotation
*Zm00001eb178890*	4	probable alpha, alpha-trehalose-phosphate synthase [UDP-forming] 10
*Zm00001eb178960*	4	ubiquitin-protein ligase/zinc ion binding protein
*Zm00001eb179000*	4	homeodomain leucine zipper protein CPHB-5
*Zm00001eb179010*	4	DEAD-box ATP-dependent RNA helicase 29
*Zm00001eb182780*	4	Bifunctional riboflavin biosynthesis protein RIBA 1 chloroplastic
*Zm00001eb183010*	4	NAP1-related protein 2
*Zm00001eb183950*	4	very-long-chain 3-oxoacyl-CoA reductase 1-like
*Zm00001eb184000*	4	cytosolic glyceraldehyde-3-phosphate dehydrogenase GAPC3
*Zm00001eb184050*	4	fasciated ear2
*Zm00001eb414730*	10	phosphopantothenate-cysteine ligase
*Zm00001eb414740*	10	DNA polymerase I A, chloroplastic
*Zm00001eb414760*	10	IST1-like protein
*Zm00001eb414780*	10	CRS2-associated factor 1 mitochondrial
*Zm00001eb414790*	10	somatic embryogenesis receptor kinase 2-like
*Zm00001eb415310*	10	rho GTPase-activating protein 1
*Zm00001eb415420*	10	ent-copalyl diphosphate synthase
*Zm00001eb415460*	10	acyl-CoA-binding protein
*Zm00001eb415690*	10	Putative MYB DNA-binding domain superfamily protein
*Zm00001eb419190*	10	probable glutathione S-transferase GSTU6
*Zm00001eb419210*	10	Glycerol-3-phosphate dehydrogenase SDP6 mitochondrial
*Zm00001eb419400*	10	acetyl-CoA carboxylase 2
*Zm00001eb420820*	10	serine carboxypeptidase 1
*Zm00001eb420890*	10	Small ribosomal subunit biogenesis GTPase RsgA 1, mitochondrial-like
*Zm00001eb420950*	10	Vacuolar protein-sorting-associated protein 11-like protein
*Zm00001eb421010*	10	splicing factor-related
*Zm00001eb421120*	10	calmodulin-binding transcription activator 4
*Zm00001eb421290*	10	dihydrolipoyllysine-residue succinyltransferase component of 2-oxoglutarate dehydrogenase complex
*Zm00001eb421340*	10	Protein auxin signaling F-box 3
*Zm00001eb421360*	10	chaperone protein ClpC1, chloroplastic
*Zm00001eb421400*	10	Protein MEI2-like 1
*Zm00001eb421870*	10	putative protein kinase superfamily protein
*Zm00001eb422170*	10	photoperiod-responsive protein

## Data Availability

The original contributions presented in this study are included in the article/Supplementary Materials. Further inquiries can be directed to the corresponding authors.

## Supplementary Materials

The following supporting information can be downloaded at https://www.mdpi.com/article/10.3390/cimb48040423/s1.
