# Supplementary material for: Mining and Analysis of Salt Tolerance Genes in Maize at the Seedling Stage
Source: Curr Issues Mol Biol. 2026 Apr 20;48(4):423. doi: 10.3390/cimb48040423 (PMC13115532; doi:10.3390/cimb48040423)
Supplement: Supplementary file 1 [file cimb-48-00423-s001.zip › Supplementary Figure.pdf]

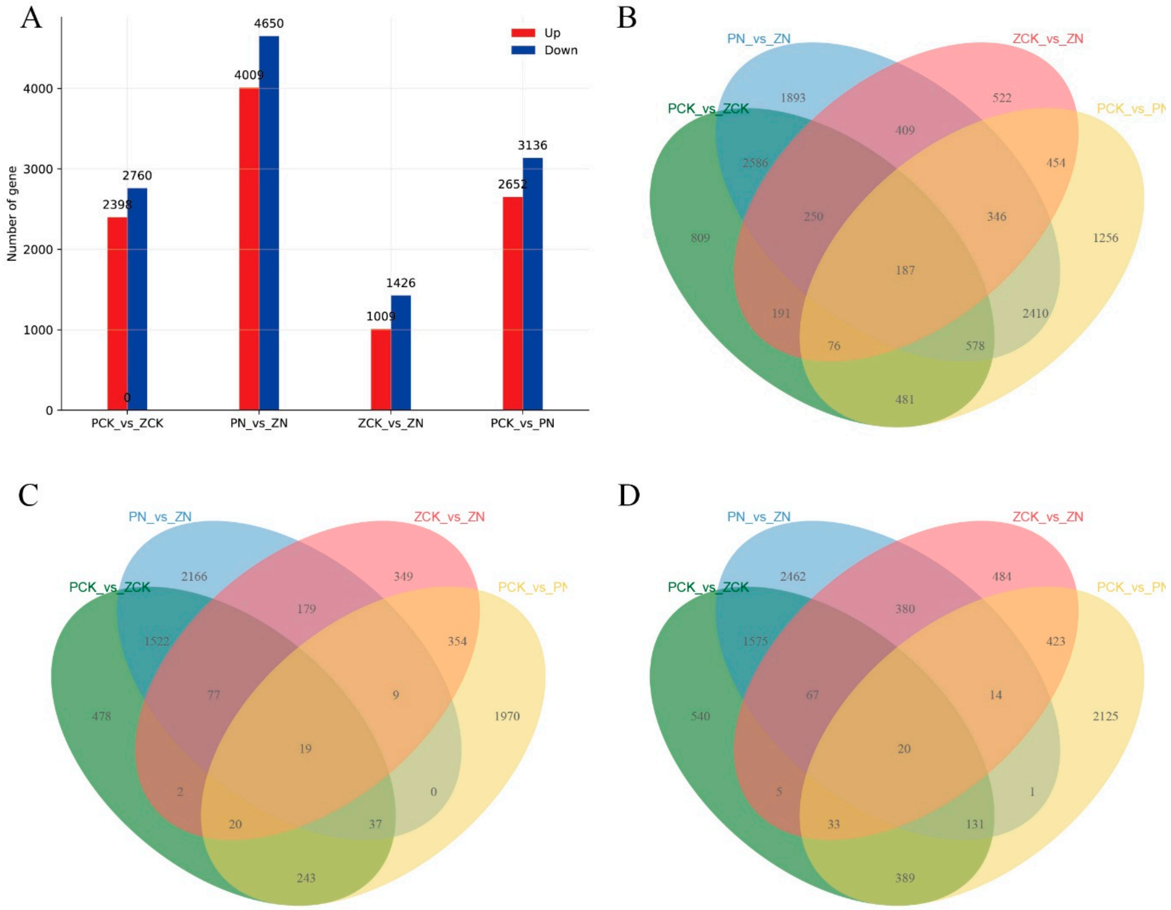

Supplementary Figure S1 Differential gene expression analysis under salt stress conditions. (**A)** Quantitative distribution of up-regulated and down-regulated DEGs across comparative groups, illustrating the magnitude of transcriptional changes. **(B-D)** Venn diagram analysis of DEG overlap: Total DEGs (B), Up-regulated DEGs (C), and Down-regulated DEGs (D), demonstrating the shared and unique stress-responsive genes between different genotype-treatment comparisons.

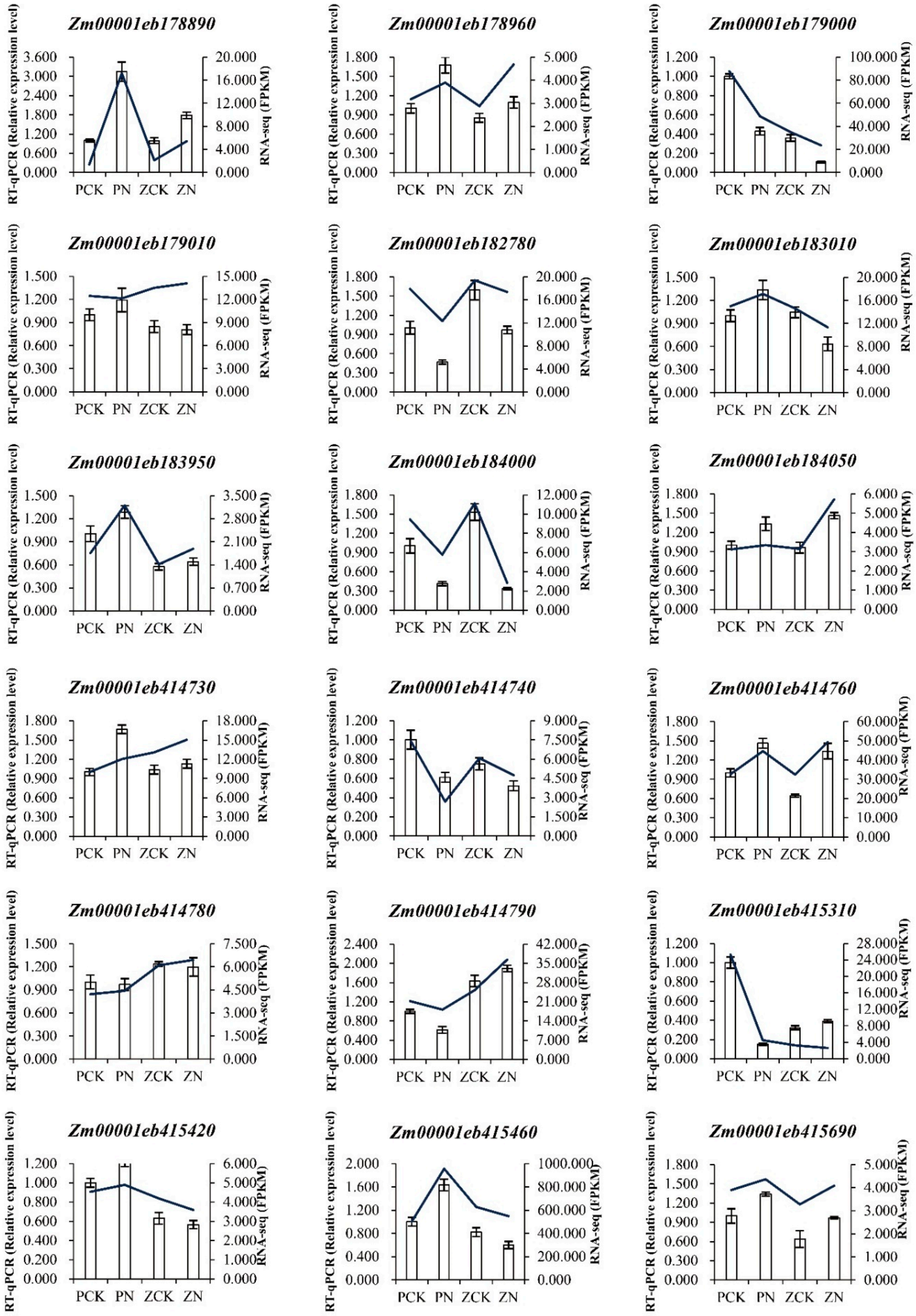
Zm00001eb178890
RT-qPCR (Relative expression level)
RNA-seq (FPKM)
0.000 0.600 1.200 1.800 2.400 3.000 3.600
0.000 4.000 8.000 12.000 16.000 20.000
PCK PN ZCK ZN
Zm00001eb178960
0.000 0.300 0.600 0.900 1.200 1.500 1.800
0.000 1.000 2.000 3.000 4.000 5.000
Zm00001eb179000
0.000 0.200 0.400 0.600 0.800 1.000 1.200
0.000 20.000 40.000 60.000 80.000 100.000
Zm00001eb179010
0.000 0.300 0.600 0.900 1.200 1.500
0.000 3.000 6.000 9.000 12.000 15.000
Zm00001eb182780
0.000 0.300 0.600 0.900 1.200 1.500 1.800
0.000 4.000 8.000 12.000 16.000 20.000
Zm00001eb183010
0.000 0.300 0.600 0.900 1.200 1.500
0.000 4.000 8.000 12.000 16.000 20.000
Zm00001eb183950
0.000 0.300 0.600 0.900 1.200 1.500
0.000 0.700 1.400 2.100 2.800 3.500
Zm00001eb184000
0.000 0.300 0.600 0.900 1.200 1.500 1.800
0.000 2.000 4.000 6.000 8.000 10.000 12.000
Zm00001eb184050
0.000 0.300 0.600 0.900 1.200 1.500 1.800
0.000 1.000 2.000 3.000 4.000 5.000 6.000
Zm00001eb414730
0.000 0.300 0.600 0.900 1.200 1.500 1.800
0.000 3.000 6.000 9.000 12.000 15.000 18.000
Zm00001eb414740
0.000 0.200 0.400 0.600 0.800 1.000 1.200
0.000 1.500 3.000 4.500 6.000 7.500 9.000
Zm00001eb414760
0.000 0.300 0.600 0.900 1.200 1.500 1.800
0.000 10.000 20.000 30.000 40.000 50.000 60.000
Zm00001eb414780
0.000 0.300 0.600 0.900 1.200 1.500
0.000 1.500 3.000 4.500 6.000 7.500
Zm00001eb414790
0.000 0.400 0.800 1.200 1.600 2.000 2.400
0.000 7.000 14.000 21.000 28.000 35.000 42.000
Zm00001eb415310
0.000 0.200 0.400 0.600 0.800 1.000 1.200
0.000 4.000 8.000 12.000 16.000 20.000 24.000 28.000
Zm00001eb415420
0.000 0.200 0.400 0.600 0.800 1.000 1.200
0.000 1.000 2.000 3.000 4.000 5.000 6.000
Zm00001eb415460
0.000 0.400 0.800 1.200 1.600 2.000
0.000 200.000 400.000 600.000 800.000 1000.000
Zm00001eb415690
0.000 0.300 0.600 0.900 1.200 1.500 1.800
0.000 1.000 2.000 3.000 4.000 5.000

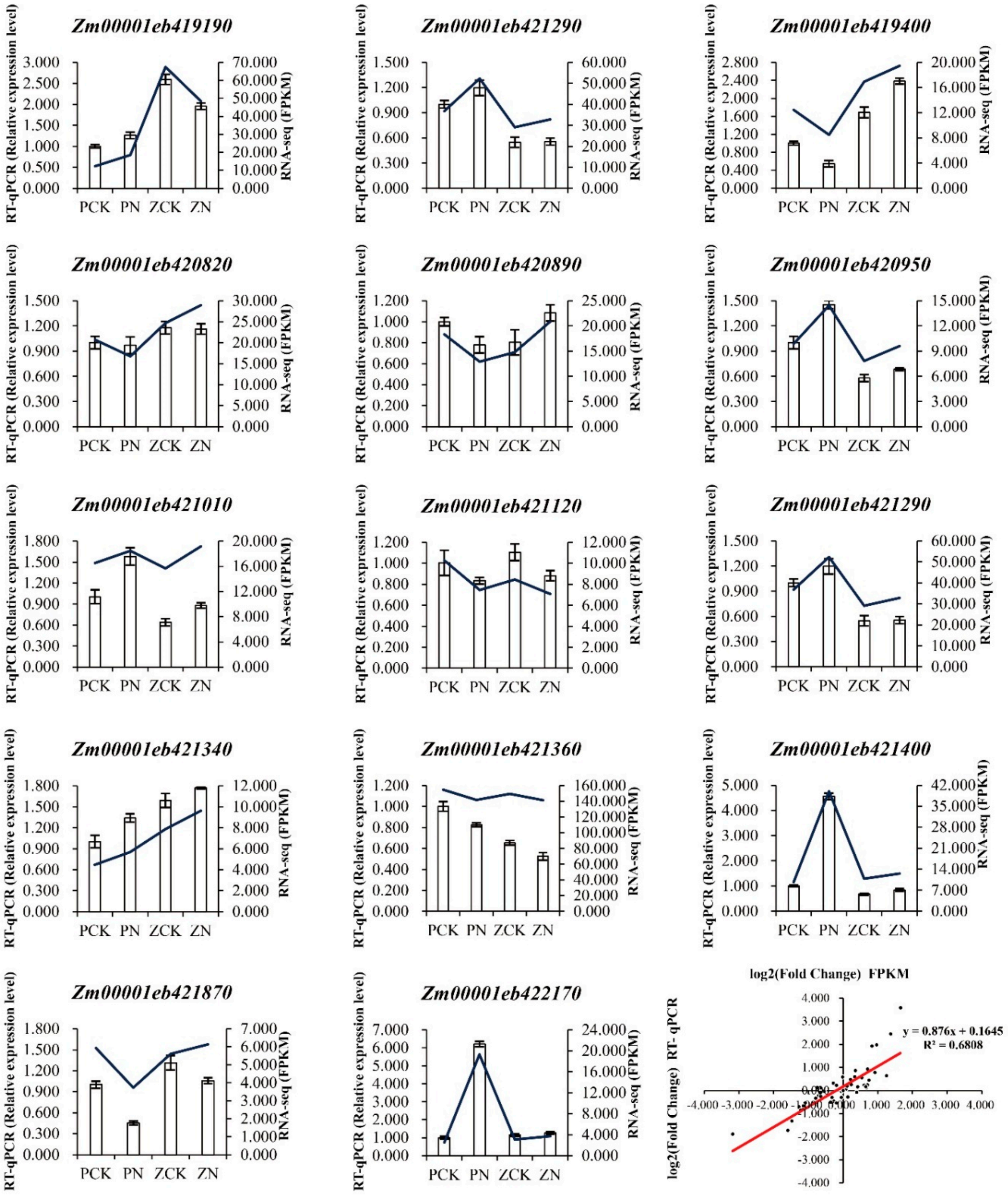

Supplementary Figure S2 Validation of DEGs by quantitative RT-PCR. Expression patterns of 32 candidate genes were verified, with normalization to the Actin reference gene. Data represent means ± SEM (n=3 biological replicates). Bar plots (left axis) show RT-qPCR results while connected lines (right axis) display corresponding RNA-seq expression levels. Treatment groups: PCK, PN, ZCK, ZN. Correlation analysis between qPCR and RNA-seq data, demonstrating high consistency between both methods.

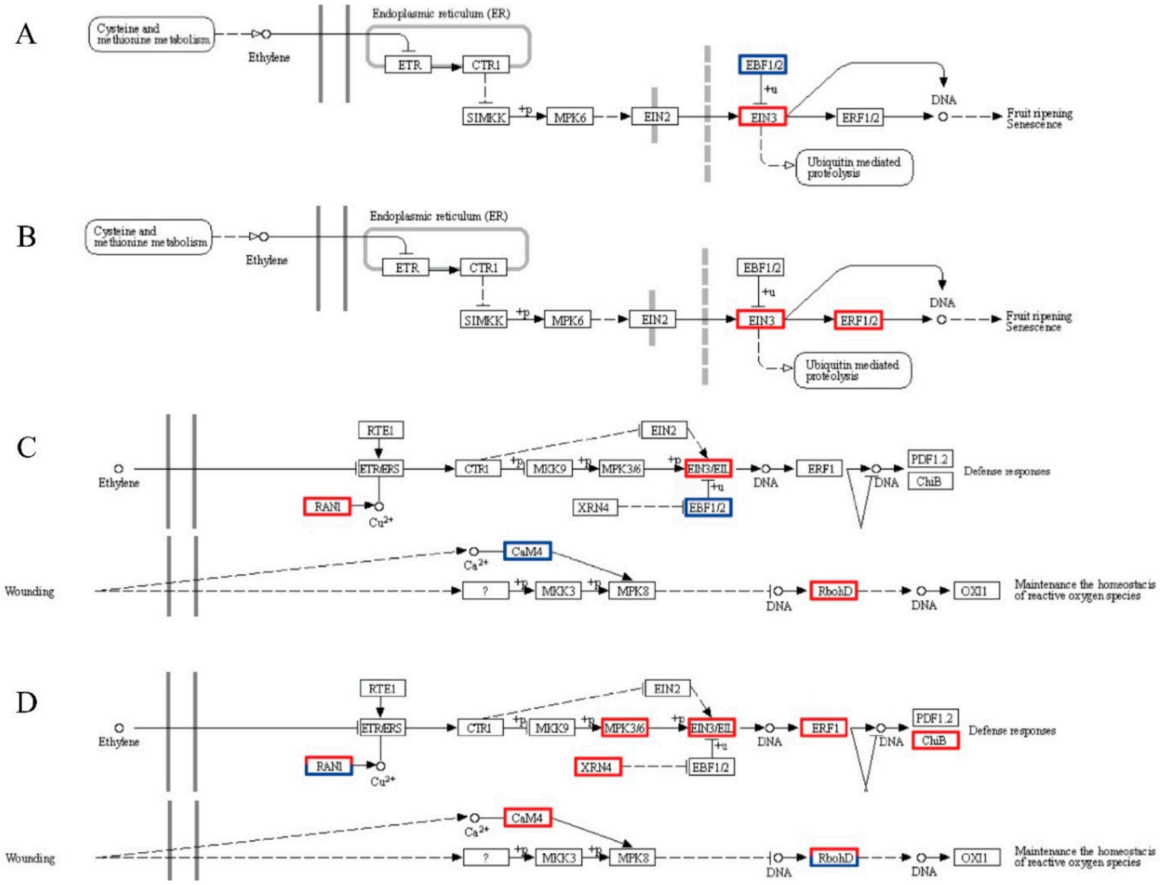

Supplementary Figure S3 Plant hormone signal transduction and MAPK signaling pathways regulating salt tolerance in different comparison groups. **(A, B)** Plant hormone signal transduction pathway. **(C, D)** MAPK signaling pathway - plant.

Note: Panels (A, C) correspond to the ZCK vs ZN comparison (Zheng58), while panels (B, D) correspond to the PCK vs PN comparison (PH4CV).
